# Dantrolene is neuroprotective in Huntington's disease transgenic mouse model

**DOI:** 10.1186/1750-1326-6-81

**Published:** 2011-11-25

**Authors:** Xi Chen, Jun Wu, Svetlana Lvovskaya, Emily Herndon, Charlene Supnet, Ilya Bezprozvanny

**Affiliations:** 1 Department of Physiology, University of Texas Southwestern Medical Center at Dallas, Dallas, Texas 75390, USA; 2 Department of Medical Physics and Bioengineering, St Petersburg State Polytechnical University, St. Petersburg, Russia; 3 Department of Pathology, University of Texas Southwestern Medical Center at Dallas, Dallas, Texas 75390, USA

**Keywords:** Huntington's disease, calcium signaling, calcium imaging, cell death, dantrolene, ryanodine receptor, aggregation, neuroprotection

## Abstract

**Background:**

Huntington's disease (HD) is a progressive neurodegenerative disorder caused by a polyglutamine expansion in the Huntingtin protein which results in the selective degeneration of striatal medium spiny neurons (MSNs). Our group has previously demonstrated that calcium (Ca^2+^) signaling is abnormal in MSNs from the yeast artificial chromosome transgenic mouse model of HD (YAC128). Moreover, we demonstrated that deranged intracellular Ca^2+ ^signaling sensitizes YAC128 MSNs to glutamate-induced excitotoxicity when compared to wild type (WT) MSNs. In previous studies we also observed abnormal neuronal Ca^2+ ^signaling in neurons from spinocerebellar ataxia 2 (SCA2) and spinocerebellar ataxia 3 (SCA3) mouse models and demonstrated that treatment with dantrolene, a ryanodine receptor antagonist and clinically relevant Ca^2+ ^signaling stabilizer, was neuroprotective in experiments with these mouse models. The aim of the current study was to evaluate potential beneficial effects of dantrolene in experiments with YAC128 HD mouse model.

**Results:**

The application of caffeine and glutamate resulted in increased Ca^2+ ^release from intracellular stores in YAC128 MSN cultures when compared to WT MSN cultures. Pre-treatment with dantrolene protected YAC128 MSNs from glutamate excitotoxicty, with an effective concentration of 100 nM and above. Feeding dantrolene (5 mg/kg) twice a week to YAC128 mice between 2 months and 11.5 months of age resulted in significantly improved performance in the beam-walking and gait-walking assays. Neuropathological analysis revealed that long-term dantrolene feeding to YAC128 mice significantly reduced the loss of NeuN-positive striatal neurons and reduced formation of Htt^exp ^nuclear aggregates.

**Conclusions:**

Our results support the hypothesis that deranged Ca^2+ ^signaling plays an important role in HD pathology. Our data also implicate the RyanRs as a potential therapeutic target for the treatment of HD and demonstrate that RyanR inhibitors and Ca^2+ ^signaling stabilizers such as dantrolene should be considered as potential therapeutics for the treatment of HD and other polyQ-expansion disorders.

## Background

Huntington's disease (HD) is an autosomal-dominant inherited neurological disorder characterized by abnormal involuntary movements (chorea, dystonia and bradykinesia) cognitive dysfunction, and psychiatric disturbance. At the molecular level, the cause of HD is a mutation in the cytosolic huntingtin (Htt) protein resulting in the expansion of a polyglutamine (polyQ)-repeat at the amino-terminus. Experimental evidence indicates that the polyQ expansion in mutant Htt (Htt^exp^) leads to a "toxic gain or loss of function", leading to the progressive and selective death of striatal medium spiny neurons (MSNs) [1,2]. However, the cellular mechanisms underlying the cause of MSN degeneration are not clear. Our previous studies demonstrated that deranged calcium (Ca^2+^) release from the endoplasmic reticulum (ER) was caused by a direct association of Htt^exp ^with the type 1 inositol 1, 4, 5- trisphosphate receptor (InsP3R1) [3], leading to apoptosis in MSNs [4]. In addition, over-expression of the cytosolic carboxy-terminus region of InsP3R1 (IC10 fragment) disrupted the Htt^exp^-InsP3R1 interaction and prevented the death of HD MSNs [5]. In more recent studies we demonstrated pathological enhancement of neuronal store-operated Ca^2+ ^entry (SOC) pathway in HD [6]. In addition, increased Ca^2+ ^influx via extrasynaptic NR2B subunit of *N*-methyl-D-aspartate receptor (NMDAR) was proposed to play an important role in excitotoxic cell death of HD MSN neurons [4,7-12]. Collectively these data indicate that Ca^2+ ^signaling plays an important role in the pathogenesis of HD [13-16].

The mechanisms of Ca^2+ ^release from intracellular stores involves several pathways, including InsP3-induced Ca^2+ ^release (IICR) mediated by the InsP3Rs and Ca^2+^-induced Ca^2+ ^release (CICR) triggered by the ryanodine receptors (RyanR). Because Ca^2+ ^release by IICR is often amplified by CICR [17] and augmented release of Ca^2+ ^from intracellular stores were toxic to HD MSNs [4,5], we reasoned that inhibiting RyanR-mediated CICR and stabilizing Ca^2+ ^signaling would have neuroprotective effects in YAC128 HD mice. We have shown previously that the RyanR antagonist and clinically relevant intracellular Ca^2+ ^stabilizer, dantrolene, was neuroprotective in mouse models of spinocerebellar ataxia type 2 (SCA2) and type 3 (SCA3) [18,19], attenuating glutamate-induced apoptosis of cultured SCA2-58Q Purkinje cells. Furthermore, feeding dantrolene to both SCA2-58Q and SCA3-YAC-84Q mice prevented neuronal cell loss and improved motor deficits [18,19]. In the present study we discovered that dantrolene pre-treatment protected cultured YAC128 MSNs from glutamate-induced apoptosis. In addition, feeding dantrolene to YAC128 mice significantly alleviated age-dependent motor deficits developed by YAC128 mice, reduced the death of NeuN-positive striatal neurons and inhibited nuclear aggregation of Htt^exp^. These results suggest that inhibiting RyanR-mediated CICR is a feasible therapeutic approach for the treatment of HD and that dantrolene is a potential agent that can be used for this purpose.

## Results

### Caffeine potentiates Ca^2+ ^release induced by glutamate in YAC128 MSNs

**Figure 1:**
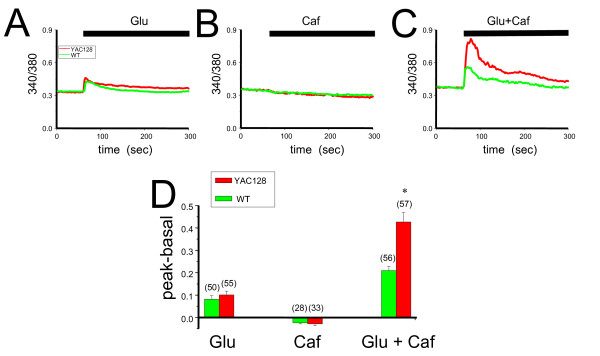
**Caffeine potentiates glutamate-induced Ca^2+ ^signals in YAC128 and WT MSN cultures**. Representative 340/380 nm Fura-2 ratio traces are shown for YAC128 (red) and WT (green) after application of 2.5 μM glutamate (*A*), 2.5 mM caffeine (*B*), and 2.5 μM glutamate + 2.5 mM caffeine simultaneously (*C*). The average differences between the peak ratio and base line ratio for each treatment was calculated and shown as mean ± SE, n = number of cells (*D*). *p < 0.05 compared to corresponding genotype treated with 2.5 μM glutamate only, ^#^*p *< 0.05 comparing YAC128 to WT, and ns = not significant using one-way ANOVA with Tukey's post test. WT, wild type; YAC, YAC128; glu, glutamate.

Previous studies demonstrated that glutamate treatment induces supranormal Ca^2+ ^responses in MSNs from HD mice [3-5,7]. To investigate whether RyanR-mediated CICR can contribute to the total Ca^2+ ^response of YAC128 MSNs to glutamate, we compared Ca^2+ ^responses induced by glutamate, the RyanR agonist caffeine, and the simultaneous application of glutamate and caffeine in WT and YAC128 MSNs at 10 days *in vitro *(DIV). The intracellular Ca^2+ ^levels were continuously monitored by Fura-2 imaging and quantitatively determined by the 340/380 nm ratio, where an increase in the ratio indicated an increase in intracellular Ca^2+ ^levels. The changes in intracellular Ca^2+ ^levels was calculated as the difference between the basal 340/380 nm value prior to glutamate and/or caffeine treatment and the peak value at the completion of the treatment in the same cell. We observed that after the application of 2.5 μM glutamate, the peak 340/380 nm value in YAC128 MSNs was not significantly different compared to WT MSNs (Figure 1A, D). Also, the addition of 2.5 mM caffeine alone did not cause a significant increase in 340/380 nm values in either YAC128 or WT MSNs (Figure 1B, D). However, when cells were treated with glutamate and caffeine together there was a significant increase in the 340/380 nm ratios as compared to glutamate treatment alone in both YAC128 and WT MSNs, suggesting that caffeine treatment can sensitize the intracellular Ca^2+ ^response to glutamate in MSNs (Figure 1C). Furthermore, the peak responses of YAC128 MSNs to glutamate and caffeine mixture were significantly higher (*p *< 0.05) than in WT MSNs (Figures 1C, D). These data demonstrate that CICR from RyanR-gated stores is significantly increased in YAC128 MSNs compared to WT when challenged with glutamate.

### Dantrolene pre-treatment protects YAC128 MSNs from glutamate-induced toxicity

**Figure 2:**
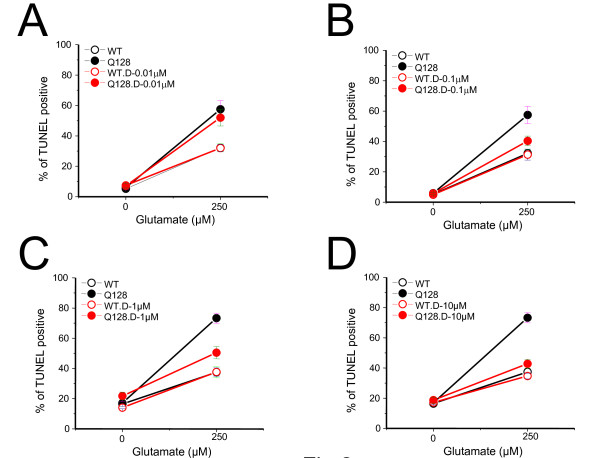
**Dantrolene protects YAC128 MSNs from glutamate-induced apoptosis**. Quantification of glutamate-induced apoptotic cell death of cultured 14DIV YAC128 (closed) and WT (open) MSNs, with (red) or without (black) 1 h dantrolene (Dan) pre-treatment. Shown are the mean ± SE of % TUNEL positive cells = (number of TUNEL positive MSNs/number of MSNs) × 100%. n = number of fields counted, Stats. WT, wild type; Q128, YAC128.

The deranged intracellular Ca^2+ ^signaling in HD MSNs is linked to increased susceptibility to glutamate-induced cell death [4-6,20-22]. Given that RyanRs appear to contribute to the augmented intracellular Ca^2+ ^levels observed in YAC128 MSNs in response to glutamate (Figure 1), we hypothesized that blockade of the RyanRs would attenuate the glutamate-induced toxicity in YAC128 MSNs. To test this hypothesis, we treated both YAC128 and WT MSNs (14DIV) with dantrolene, a blocker of RyanR activity, prior to the application of 250 μM glutamate, a concentration shown to induce MSN apoptosis in an *in vitro *model of HD [4-6,20-22]. As we reported previously [4-6,20-22], in the absence of glutamate, there was no significant difference between the percentage of apoptotic neurons in YAC128 and WT MSN cultures (Table 1). After exposure to 250 μM glutamate, the percentage of apoptotic neurons in YAC128 MSN cultures was increased significantly when compared to WT cultures (Table 1, Control 1 and Control 2 groups). To assess the neuroprotective effects of dantrolene, MSNs were pretreated for 1 h with various concentrations of dantrolene (10 nM - 50 μM range) and after the application of 250 μM glutamate, the percentage of apoptotic neurons was determined by terminal deoxynucleotidyl transferase dUTP nick end labeling (TUNEL). We found that 0.01 μM dantrolene had no significant effects on the glutamate-induced apoptosis of YAC128 MSNs (Figure 2A, Table 1); 0.1 μM and 1 μM dantrolene showed partial rescue of YAC128 MSNs treated with glutamate (Figure 2B, C, Table 1); and 10 μM and 50 μM dantrolene was able to prevent most YAC128 MSNs from glutamate-induced toxicity (Figure 2D, Table 1). Interestingly, dantrolene treatment was not able to rescue WT MSNs from glutamate-induced toxicity (Figure 2, Table 1), suggesting that RyanRs do not play a significant role in the glutamate-induced apoptosis of WT cultures. These data demonstrate that the CICR Ca^2+ ^release from RyanRs play a significant and specific role in mediating the increased glutamate-induced cell death observed in YAC128 MSNs and that dantrolene treatment is effective in protecting YAC128 MSNs from glutamate toxicity.

**Table 1:** Effects of dantrolene on glutamate-induced apoptosis in WT and YAC128 MSN.

Dantrolene treatment	WT (% TUNEL-positive)		YAC128 (% TUNEL-positive)	
	
	0 μM glutamate	250 μM glutamate	0 μM glutamate	250 μM glutamate
Control 1	5.20 ± 1.16	32.22 ± 2.66	5.94 ± 1.11	57.45 ± 5.62
0.01 μM	7.45 ± 1.08	32.02 ± 2.28	7.13 ± 1.37	52.03 ± 5.45
0.1 μM	4.92 ± 1.48	31.36 ± 3.64	5.73 ± 0.87	40.37 ± 3.18*
				
Control 2	16.43 ± 1.25	37.43 ± 3.66	17.05 ± 1.18	73.26 ± 3.32
1.0 μM	14.05 ± 0.68	37.61 ± 2.75	21.76 ± 2.60	50.45 ± 4.11***
10 μM	17.22 ± 1.39	34.69 ± 1.86	18.75 ± 1.92	42.79 ± 2.85***
50 μM	17.94 ± 0.95	38.20 ± 2.21	19.73 ± 1.67	35.85 ± 2.24***

***p < 0.001 compared to Control 2, *p < 0.05 compared to Control 1 using one-way ANOVA with Tukey's post test.

### Dantrolene rescues the motor coordination deficiencies in YAC128 mice

Dantrolene can be considered a general Ca^2+ ^signaling stabilizer. The neuroprotective characteristics of dantrolene have been shown in glutamate toxicity assay with YAC128 MSNs (Figure 2, Table 1) and in previously reported experiments with cultured neuronal cells and animal models of excitotoxicity [23-31]. We recently reported that dantrolene was neuroprotective in mouse models of SCA2 and SCA3 [18,19]. These results lead us to evaluate the potential beneficial effects of dantrolene in the YAC128 HD mouse model. For these studies, 5 mg/kg of dantrolene in phosphate buffered saline (PBS) and corn flour was orally delivered to YAC128 and WT mice twice a week from 2 months of age until all the mice were 11.5 months of age, followed by a washout period of 2 months (13.5 months of age). The control groups of WT and YAC128 mice were fed with PBS and corn flour alone. Behavioral assessment of all four groups of mice was performed using the "beam-walk" assay. Basal beam-walking performance was determined before the initiation of dantrolene feeding at 2 months of age. Beam-walking performance data were collected at 2, 4, 7, 9.5, 11.5, and 13.5 (washout) months of age. As in our previous studies with YAC128 HD mice [5,32,33], three kinds of beams (17 mm round, 11 mm round, and 5 mm square) were used for testing. The "latency" and "number of foot slips" were for each mouse determined on each beam.

**Figure 3:**
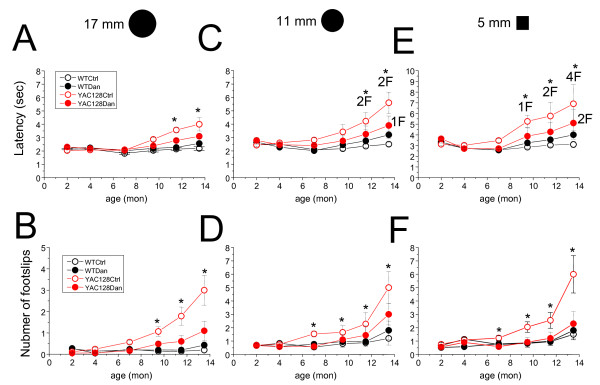
**Dantrolene feeding improves performance of YAC128 mice in the beam-walk test**. Motor coordination performance of WT and YAC128 mice in dantrolene trial. A-F. Beam walk assay. The average time to cross the beam (*A, C, E*) and the average number of foot slips on the beam (*B, D, F*) are shown for beam-walking experiments performed with 17 mm round plastic beam (*A, B*), 11 mm round plastic beam (*C, D*), and 5 mm square wood beam (*E, F*). The data for WT control mice (open black circles), YAC128 control mice (open red circles), WT mice fed with dantrolene (filled black circles), and YAC128 mice fed with dantrolene (filled red circles) are shown as mean ± SE (for the numberof mice (10) in each experimental group, see Table 2) 2, 4, 7, 9.5, 11.5, and 13.5 (washout) months of age. While counting the foot slips of the mice with "crawling behavior, " we considered every step as one foot slip. *p < 0.05, significant differences between control WT group and control SCA3 group. "*x*F" on C and E mouse fell off the beam and failed the test where *x *= number of mice. *p < 0.05 using one-way ANOVA with Tukey's post test. WT, wild type; YAC, YAC128; Ctrl, control; Dan, dantrolene.

Consistent with our previous findings [5,32,33], we found that with age the PBS-fed group of YAC128 mice exhibited a progressive impairment in beam-walking ability, resulting in longer beam traverse latencies and increased number of foot slips, compared to the WT PBS-fed group (Figure 3). These impairments in YAC128 mice became more evident as beam difficulty increased (Figure 3). Significant differences (*p *< 0.05) between the beam-walking performance of control YAC128 and control WT groups were observed at 11.5 and 13.5 months of age on the 17 mm round beam (Figure 3B); at 7, 9.5, 11.5 and 13.5 months of age on 11 mm round beam (Figure 3C, D) and at 7, 9.5, 11.5 and 13.5 months of age 5 mm square beam (Figure 3E, F). Dantrolene feeding had no significant effect on the beam-walk performance of the WT mice (Figure 3, black data points). However, feeding dantrolene to YAC128 mice improved their beam-walking performance significantly (*p *< 0.05) by shortening the beam traverse latencies and decreasing the number of foot slips (Figure 3, red data points). Significant differences (*p *< 0.05) in latency between the dantrolene-fed YAC128 group and the YAC128 control group were detected at 11.5 and 13.5 months of age on 17 mm round beam (Figure 3A) and 11 mm round beam (Figure 3C), and at 9.5, 11.5, 13.5 months of age on 5 mm square beam (Figure 3E). At 7, 9.5, 11.5, and 13.5 months of age, the number of foot slips of dantrolene-fed YAC128 mice on all three beams were very similar to control WT mice (Figure 3B, D, F). We noticed that, after dantrolene withdrawal, the performance of both WT and YAC128 groups of mice was impaired, with increased latencies and elevated numbers of foot slips (Figure 3). This phenomenon is most likely to be attributed to the removal of the sedative effect of dantrolene, which resulted in the increased anxiousness of the mice. Despite this "withdrawal effect" the dantrolene-fed YAC128 mice still performed significantly better than control YAC128 mice in most tasks 13.5 months of age (Figure 3). While conducting beam-walking assays, we observed that some aging mice exhibited periods of "crawling behavior", defined as prolonged contact between the thorax and abdomen of the mice and beam surface, with the mice using their fore-limbs to drag themselves along the beam. One mouse in the YAC128 control group crawled on the 11 mm round beam at 11.5 and 13.5 months of age and one mouse in the YAC128 control group crawled on 5 mm square beam at 13.5 months of age. Some mice fell off of the beam during testing. Two mice in the YAC128 control group fell off of the 11 mm round beam when tested at 11.5 and 13.5 months of age (Figure 3C). For the test on 5 mm square beam, one mouse fell off at 9.5 months of age, 2 mice fell off at 11.5 months of age, and 4 mice fell off at 13.5 months of age (Figure 3E). In contrast, none of the mice in the WT groups and the dantrolene-fed YAC128 group exhibited crawling behavior or fell off the beams at any age tested. The incidence of crawling behavior was consistent with the general performance of these mice in beam-walk assay, with the YAC128 control group performing the worst, dantrolene-fed YAC128 group performing better, and the WT groups performing the best.

**Figure 4:**
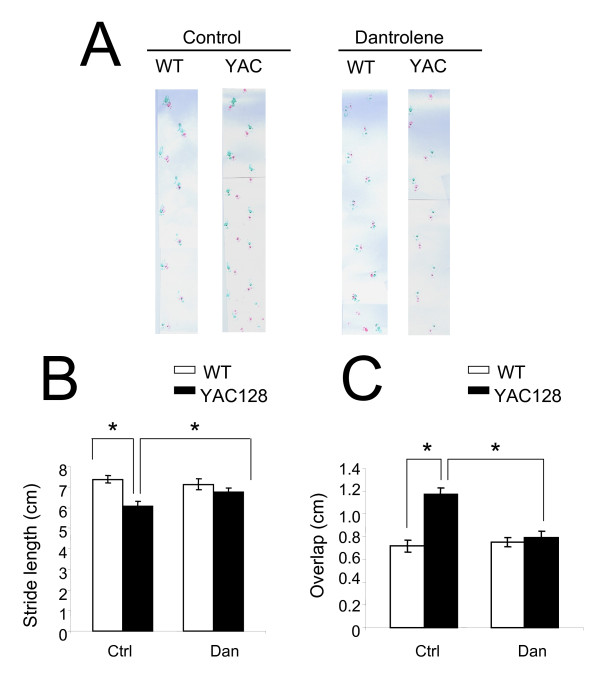
**Dantrolene feeding improves gait abnormalities in YAC128 mice**. Gait analysis of WT and YAC128 mice in dantrolene trial. (*A*). The footprint patterns of 13.5-month-old WT and YAC128 for control and dantrolene groups are shown. (*B*) Stride lengths (cm) and (*C*) front/hind footprint overlaps (cm) of WT and YAC128 mice in dantrolene trial are shown as mean ± SE (n = number of mice (10) in each groupsee Table 2), *p < 0.05 using one-way ANOVA with Tukey's post test. WT, wild type; YAC, YAC128; Ctrl, control; Dan, dantrolene.

At the conclusion of the beam-walking behavioral experiments (13.5 months of age), we assessed gait abnormalities in the four groups of mice by footprint pattern analysis (Figure 4). The footprint patterns were assessed quantitatively by the measurements of stride length and front/hind footprint overlap as we previously described for YAC128 HD mice [5,32,33]. Consistent with our previous findings [5,32,33], the stride length of YAC128 PBS-fed mice was significantly (*p *< 0.05) shorter than WT PBS-fed mice (Figure 4A, B). Dantrolene feeding significantly increased the stride length of YAC128 mice, but had no obvious effects on stride length of WT mice (Figure 4A, B). We further found that front/hind footprint overlap was significantly higher in YAC128 control group than in WT control group and feeding dantrolene to YAC128 mice alleviated the footprint overlap deficit (Figure 4A, C). These data demonstrate that dantrolene feeding can rescue the motor coordination deficits observed in YAC128 mice, suggesting that striatal function is regained and/or preserved by dantrolene.

### Dantrolene feeding reduces NeuN-postitive cell loss in striata of YAC128 mice

**Figure 5:**
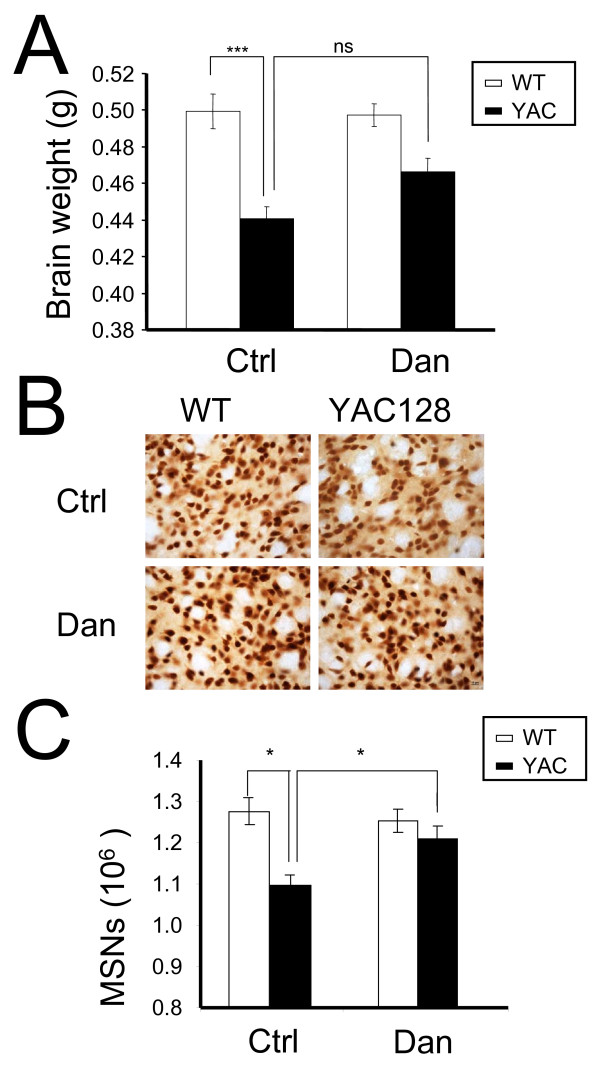
**Dantrolene feeding reduces NeuN-positive cell loss in YAC128 striata**. (*A*) The brain weight of control (PBS-fed) and dantrolene-fed WT and YAC128 mice was measured at 14 months of age after 4% paraformaldehyde perfusion. The brain weight is shown as mean ± SE (g) (n = number of mice (10) in each groupsee Table 2). (*B*) The striatal slices from 14 months old control (PBS-fed) and dantrolene-fed WT and YAC128 mice were stained by the neuronal nuclear marker NeuN. Representative images are shown. (*C*) Striatal cell counts obtained as a result of stereological analysis of NeuN-stained striatal slices from 14 months old control (PBS-fed) and dantrolene-fed WT and YAC128 mice. The MSN numbers are shown as mean ± SE (n = number of mice, see Table 2). ***p < 0.001, *p < 0.05 using one-way ANOVA with Tukey's post test. WT, wild type; YAC, YAC128; Ctrl, control; Dan, dantrolene.

To further evaluate the potential neuroprotective effects of dantrolene in the striata of the experimental mice we determined brain weight and counted MSNs by an unbiased stereology approach. Our analysis was focused on the striatal region because it is severely affected in HD patients and we show that dantrolene treatment is neuroprotective for YAC128 MSNs *in vitro *(Figure 2, Table 1). At the conclusion of behavioral analysis (14 months of age), mice from all four groups were perfused transcardially with 4% paraformaldehyde in PBS and the brains were extracted and weighed. We found that the brains of control YAC128 mice weighed significantly (*p *< 0.05) less than the brains of control WT mice (Figure 5A). Feeding dantrolene to WT mice had no significant effect on the brain weight of these mice (Figure 5A, Table 2). However, feeding dantrolene to YAC128 mice increased brain weight compared to control YAC128, though the difference was not statistically significant (Figure 5A, Table 2). Importantly, long-term feeding of dantrolene did not induce general toxicity in WT or YAC128 mice by pathological assessment of muscle, heart and liver tissues (Table 3).

**Table 2:** Brain weight and MSN counts of 14 months old WT and YAC128 mice.

Group name	Number of female mice	Brain weight (g)	**MSN counts (10**^**6**^**)**
WT-Ctrl	10	0.499 ± 0.01	1.27 ± 0.03

WT-Dan	10	0.495 ± 0.01	1.263 ± 0.03

YAC128-Ctrl	10	0.437 ± 0.01***	0.997 ± 0.03*

YAC128-Dan	10	0.461 ± 0.01	1.18 ± 0.030^#^

The brain weight and MSN counts from NeuN staining and stereological analysis are shown as mean ± SE. ***p < 0.001, *p < 0.05 compared to WT-Ctrl and ^#^*p *< 0.05 compared to YAC128-Ctrl using one-way ANOVA with Tukey's post test.

Pathological analysis of WT and YAC128 mice tissues following dantrolene trial.

**Table 3:** Pathological analysis of WT and YAC128 mice tissues following dantrolene trial.

Group name	Number of mice analyzed	No. of mice per group with any degree of inflammation		
		
		muscle	heart	liver
**WT Ctrl**	6	0	0	6

**YAC128 Ctrl**	4	0	1	4

**WT Dan**	3	0	0	3

**YAC128 Dan**	6	0	1	6

To obtain quantitative information about neuronal loss in the experimental mice, we stained the striatal slices for NeuN, a nuclear-specific marker of mature neurons (Figure 5B), and performed stereological analysis. NeuN-positive neurons in each striatum were counted blindly with respect to the nature of slices (genotype and drug treatment). Consistent with our previous findings [5,32,33], we determined that PBS-fed YAC128 mice showed significant neuronal loss in the striatum (*p *< 0.05) when compared with PBS-fed WT mice (Figure 5C, Table 2). We further found that dantrolene feeding had no significant effect on NeuN-positive counts in the striata of WT mice but significantly increased NeuN-positive counts (*p *< 0.05) in YAC128 mice (Figure 5C, Table 2). The data show that dantrolene feeding protected against the age-dependent loss of NeuN-positive neurons in the striata of YAC128 mice.

### Dantrolene feeding inhibits the aggregation of Htt^exp ^in the striata of YAC128 mice

**Figure 6:**
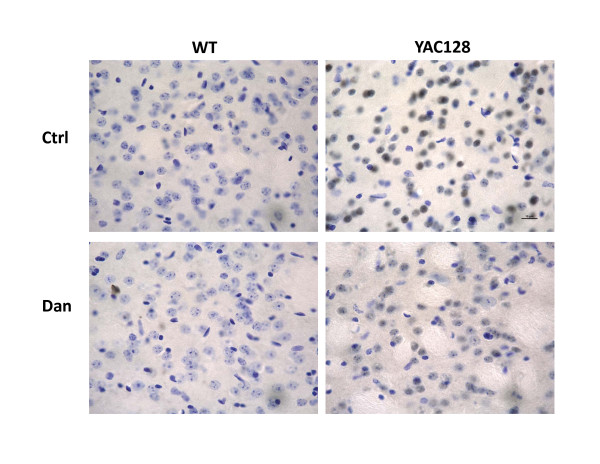
**Dantrolene feeding inhibits Htt^exp ^aggregation in YAC128 striatal cells**. The striatal slices from 14 months old control (PBS-fed) and dantrolene-fed WT and YAC128 mice were stained with an anti-Htt monoclonal antibody (dark grey). Nuclei were counter-stained with cresyl violet (blue). Representative images of 3 mice are shown. WT, wild type; YAC, YAC128; Ctrl, control; Dan, dantrolene.

Given that Htt^exp ^aggregation is thought to induce the progressive and selective death of striatal MSNs [1,2], we wanted to determine if dantrolene feeding could prevent or inhibit the aggregation of Htt^exp ^and provide a potential mechanism of neuroprotection in YAC128 mice. Using brain slices from mice representing all 4 treatment groups (Figure 5, Table 2), we stained for aggregated Htt^exp^, where positive staining for aggregated Htt^exp ^appears dark gray due to the nickel in the detection medium. Nuclei were counterstained with cresyl violet. Consistent with previous findings [34], PBS-fed YAC128 mice showed significant Htt^exp ^aggregation in striatal cells, indicated by dark and condense staining in the nucleus, as compared to PBS-fed WT (Figure 6). Interestingly, dantrolene-fed YAC128 mice showed a weaker and distributed staining, which suggests that dantrolene dramatically reduced the degree of Htt^exp ^aggregation in striatal cells as compared to PBS-fed YAC128. Striatal cells from dantrolene-fed WT mice appeared similar to cells from control mice (Figure 6). These observations suggest that dantrolene feeding may significantly reduce the amount of Htt^exp ^nuclear aggregation in striatal neurons of YAC128 mice.

## Discussion

In our previous studies we demonstrated that abnormal neuronal Ca^2+ ^signaling plays a significant role in the pathogenesis of HD and other polyQ expansion disorders [3-6,18-20,32,35]. We have also found that the Ca^2+ ^stabilizer and RyanR antagonist, dantrolene, was neuroprotective in the mouse models of polyQ expansion disorders SCA2 and SCA3 [18,19]. The data in the present study point to the role of RyanRs in the exaggerated Ca^2+ ^response to glutamate observed in MSNs from the YAC128 model of HD (Figure 1). Furthermore, we demonstrated that the treatment of RyanR inhibitor dantrolene significantly protected cultured YAC128 MSNs from glutamate-induced apoptosis (Figure 2). Consistent with our *in vitro *findings, we showed that long-term feeding of dantrolene to YAC128 mice significantly (*p *< 0.05) alleviated age-related motor coordination deficits (Figure 3, 4) and prevented NeuN-positive neuronal loss (*p *< 0.05) (Figure 5, Table 2) while leaving WT control mice and neurons intact. Finally, dantrolene-fed YAC128 mice showed less Htt^exp ^nuclear aggregation in striatal cells compared to control YAC128 mice (Figure 6), further supporting neuroprotective effects of dantrolene treatment. These data provide further support for the significant role of disturbed intracellular Ca^2+ ^signaling in the pathogenesis of polyQ expansion diseases and offer a potential, and clinically relevant, drug for the treatment of HD.

Our laboratory has elucidated the interaction between Htt^exp ^and InsP3R1 as well as other Ca^2+^-related mechanisms relevant to HD (for reviews, see [13-16]). Based on these results we argued that the modulation of intracellular Ca^2+ ^signaling is a viable approach in our pursuit of disease-modifying therapies for HD. We first discovered that Htt^exp ^binds directly and specifically to the C-terminus region of InsP3R1 and that this interaction is important for the augmented activity of the receptor in planar lipid bilayers and in cultured MSNs [3]. We then confirmed that MSNs from YAC128 HD mice had disturbed IP3R-mediated Ca^2+ ^signaling which lead to glutamate-induced apoptosis in YAC128 MSNs [4]. In a more recent study we demonstrated pathological enhancement of neuronal store-operated Ca^2+ ^entry (SOC) pathway in HD [6]. In addition, increased Ca^2+ ^influx via extrasynaptic NR2B subunit of NMDAR was proposed to play an important role in excitotoxic cell death of HD MSN neurons [4,7-12]. The CICR mechanism supported by RyanR can amplify the signal from other Ca^2+ ^sources such as InsP_3_R1 and NMDARs. Perhaps the effectiveness of dantrolene to reduce or block glutamate-induced apoptosis in YAC128 MSN cultures (Figure 2, Table 1) is attributed to the fact that by inhibiting the RyanRs alone, both glutamate signaling pathways important for inducing toxicity are blunted at the same time, leading to robust neuroprotection. In addition to RyanR, dantrolene may also inhibit neuronal SOC pathway [36], which may also contribute to neuroprotective effects of dantrolene observed in our experiments [6].

There are drugs available to help manage the clinical symptoms of HD but currently there is no treatment to stop or reverse the course of the disease. The only drug approved by the US Food and Drug Administration to specifically treat HD is tetrabenazine (TBZ), an inhibitor of the vesicular monoamine transporter that alleviates chorea. Therefore, a notable finding of our study is that long-term feeding of dantrolene to YAC128 mice was able to prevent neurodegeneration in the striatum (Figure 5), reduced accumulation of Htt^exp ^muclear aggregates (Figure 6) and significantly improve the motor coordination deficiencies (Figure 3, 4) that are characteristic to this model and HD pathogenesis. In motor coordination assays the benefits of dantrolene feeding to YAC128 mice were similar to the benefits of TBZ feeding that we evaluated previously [32,33]. Thus, it can be expected that dantrolene or other similar RyanR inhibitors may result in significant clinical benefits for HD patients.

Many studies have brought to light the neuroprotective properties of dantrolene (for recent review, see [37]). For example, in cultured cortical neurons, dantrolene reduced the glutamate-induced increases in intracellular Ca^2+^-concentrations by 70% under physiologic conditions, and protected against glutamate-induced neurotoxicity [23]. The short-term delivery of dantrolene has been demonstrated previously to be neuroprotective in acute excitotoxicity paradigms, such as cerebral ischemia [26,38] and a kainic acid injection model [24,28,30]. It has also been demonstrated that dantrolene can prevent apoptosis in neurons by ameliorating poly(ADP-ribose) polymerase (PARP)-related bioenergetic failure during DNA repair in rat brain [39] and up-regulating anti-apoptosis proteins such as Bcl-2 in different cell lines [40,41]. We have previously demonstrated the beneficial effects of long-term dantrolene treatment in SCA2 and SCA3 genetic mouse models [18,19]. Data from such studies has inspired researchers to consider the utility of dantrolene as treatment for many neurodegenerative diseases, including Alzheimer disease (AD) [42,43], amyotrophic lateral sclerosis [44], and ataxia [18,19] (for recent review [45]). However, in our experiments we found that long-term feeding of dantrolene to a mouse model of AD actually exacerbated the neuropathology related to the disease [46]. Dantrolene has been used safely in the clinic for decades as treatment for malignant hyperthermia, but is also used to manage neuroleptic malignant syndrome, muscle spasticity, 3, 4-methylenedioxy methylamphetamine ("ecstasy") intoxication, serotonin syndrome, and 2, 4-dinitrophenol poisoning [47]. However, dantrolene can also induce significant side effects, ranging from transient muscle weakness to more severe respiratory failure [47] in both acute and chronic treatment regimens. In our experiments, we saw a neuroprotective effect at 5 mg/kg, which is comparable to the effective dose of 2.4 mg/kg in healthy volunteers who experienced dizziness and muscle weakness [47]. We have not observed significant toxic effects of dantrolene after pathological examination of skeletal mucle, heart and liver of WT and YAC128 mice fed with dantrolene (Table 3).

## Conclusions

Obtained results provide further support to the "Ca^2+ ^hypothesis of HD" [13-16] and highlight similarities in the pathogenesis of HD, SCA2 and SCA3 [18,19,48,49]. Our data also implicate RyanRs as a potential therapeutic target for the treatment of these disorders. Moreover, our results indicate that RyanR inhibitors and Ca^2+ ^signaling stabilizers such as dantrolene should be considered as potential therapeutics for the treatment of HD and other polyQ-expansion disorders. It is not clear however if dantrolene has a safety profile that allows for its long-term use in clinic. The effect of chronic low dose administration of dantrolene in humans is not known and further study is required to determine if dantrolene is an appropriate clinical tool for HD and other polyQ expansion disorders.

## Methods

### Primary medium spiny neuron (MSN) cultures

All animal studies were approved by the University of Texas Southwestern Medical Center Animal Care and Use Committee. YAC128 transgenic mice (FVBN/NJ background strain) were obtained from Jackson Labs (stock number 004938) and breeding of YAC128 mice was previously described [50]. Briefly, heterozygous male YAC128 mice were crossed with the wild type (WT) female mice and the resulting litters were collected on postnatal days 1-2. The pups were genotyped by PCR with primers specific for exons 44 and 45 of human *HTT *and MSN cultures of WT and YAC128 mice were established as described previously [4,7,20,32]. Briefly, cultures were grown on poly-L-lysine (Sigma) coated 12 mm round coverslips (Assistent) in Neurobasal-A medium supplemented with 2% B27, 1 mM L-glutamine and penicillin-streptomycin (Invitrogen). Cultures were incubated at 37°C in a 5% CO_2 _environment.

### Ca^2+ ^imaging experiments

Fura-2 Ca^2+ ^imaging experiments were performed as previously described [4]. The MSN cultures were maintained until 10 days *in vitro *(DIV) and then loaded with 5 μM Fura-2AM (Invitrogen) in artificial cerebral spinal fluid (aCSF) (140 mM NaCl, 5 mM KCl, 1 mM MgCl_2_, 2 mM CaCl_2 _and 10 mM HEPES at pH 7.3) for 45 min at 37°C. For imaging experiments, the coverslips were mounted onto a recording chamber (RC-26G; Warner Instruments) that was maintained at 37°C (PH1; Warner Instruments) and positioned on an Olympus IX-70 inverted microscope. Before measurements were taken, the coverslips were washed extensively with Ca^2+^-free aCSF (CaCl_2 _was replaced with 100 μM EGTA). For Ca^2+ ^imaging experiments, the MSN cells were intermittently excited by 340 and 380 nm UV light using DeltaRAM illuminator (Photon Technology International (PTI)), and the 510 nm emitted light was collected by an IC-300 camera (PTI). Images were collected using a 60× UV-grade oil immersion objective (Olympus), then digitized and analyzed by ImageMaster Pro software (PTI). Baseline (1 min) measurements were obtained before bath application of 2.5 μM glutamate and/or 2.5 mM caffeine dissolved in Ca^2+^-free aCSF. Images at 340 and 380 nm excitation wavelengths were captured every 5 s and shown as 340/380 image ratios at the time points as indicated. Background fluorescence was determined according to the recommendations of the manufacturer (PTI) and subtracted.

### *In vitro *cell death assay

The *in vitro *cell death assay was performed as described previously for MSN cultures [4,5,20]. Dantrolene was added to the 14 DIV MSN cultures at 0.0, 0.1, 1, 10 or 50 μM. After 1 h incubation, 250 μM glutamate in Neurobasal-A was added to the culture medium and MSNs were maintained in a cell culture incubator (humidified 5% CO2, 37°C) for 7 h. Immediately after glutamate treatment, neurons were fixed for 30 min in 4% paraformaldehyde plus 4% sucrose in PBS (pH 7.4), permeabilized for 5 min in 0.25% Triton X-100, and assayed for apoptosis using the DeadEnd Fluorometric TUNEL System (Promega) according to manufacturer's instructions. Nuclei were counterstained with 5 μM propidium iodide (PI) (Molecular Probes). Coverslips were washed with PBS and mounted in Mowiol 4-88 (Polysciences). Fluorescein isothiocyanate (FITC)- and PI-fluorescent images were collected with an Olympus IX70 microscope using a 40× objective, a Cascade: 650 camera (Roper Scientific), and METAFLUOR software (Universal Imaging, Downingtown, PA). Four to six randomly chosen microscopic fields containing 200-300 MSN were captured. The percentage of TUNEL-positive cells was determined by taking the cell number of TUNEL-positive MSNs and dividing by the total number of MSNs (or PI stained neurons) × 100%. The percentage of TUNEL-positive cells was among all the fields per treatment was averaged and presented as the mean ± SE (n = number of fields counted). The nuclei of glial cells, identified by their large size and weak PI staining, were not counted in the analysis.

### Drug delivery in mice

Dantrolene was fed to mice as we described previously in a tetrabenzine trial in YAC128 mice [33] and dantrolene trials in SCA3-YAC-84Q [19] and SCA2-58Q mice [18]. Briefly, groups of 10 WT mice and 10 YAC128 mice were fed 100 μg of dantrolene suspended in 50 μl of PBS with 2% corn flour resulting in a dosage of 5 mg/kg. The control groups (10 WT and 10 YAC128) were fed with 2% corn flour in PBS. All mice were fed orally twice per week from 2 to 11.5 months of age.

### Motor coordination assessments in mice

The motor coordination experiments were performed as we described previously in a tetrabenzine trial in YAC128 mice [33] and dantrolene trials in SCA3-YAC-84Q [19] and SCA2-58Q mice [18]. The "beam-walking" assay was performed using a homemade experimental setup. The 17 mm round plastic beam, 11 mm round plastic beam, and 5 mm square wood beam were used in our studies. At each time point, the mice were trained on the beams for 3 consecutive days (four trials per day) to traverse the beam into the enclosed box. Once a stable baseline of performance was obtained, the mice were tested in three consecutive trials on the 17 and 11 mm round plastic beams and the 5 mm square wood beam, progressing from the widest to the narrowest beam. The latency to traverse the middle 80 cm of each beam and the number of times the hind-paws slipped off each beam were recorded for each trial. The mean scores for each beam at a particular time point were used in the analysis. For the footprint test, the forepaws and hindpaws of the mice were coated with purple and green non-toxic paints, respectively. The mice were trained to walk along a paper-covered runway that was 50 cm long, 10 cm wide, with 10 cm high walls into an enclosed box. All the mice were given three runs per day for 3 consecutive days. A fresh sheet of white paper was placed on the floor of the runway for each run. The footprint patterns were assessed quantitatively by the measurements of stride length and fore-paw/hind-paw overlap as we described previously [19,32].

### Neuropathological assessments in mice

The neuropathological assessments were performed as previously described [19,32]. After all the behavioral tests (14.5 month time point), the mice were terminally anesthetized by ketamine/xylazine and perfused transcardially with PBS then 4% paraformaldehyde in 0.1 M PBS, pH 7.4 as before [19,32]. All brains were removed, weighed, transferred to post-fixative (4% paraformaldehyde) for 24 h at 4°C, and equilibrated in 20-30% (w/v) sucrose in PBS for 24-48 h 4°C. The brains were processed and cut into 30 μm-thick coronal sections using a SM2000R sliding microtome (Leica, Bannockburn, IL). Coronal sections spaced 360 μm apart throughout the striatum (in the range from +1.70 mm to -2.30 mm relative to bregma) were collected. For the quantification of neuronal loss, sections were stained with anti-NeuN monoclonal antibody (1:1000 dilution; Millipore, Billerica, MA). Biotinylated anti-mouse IgG reagent was used as the secondary antibody (1:250 dilution; M.O.M. kit, Vector Laboratories). Signal was amplified with an ABC Elite kit (Vector Laboratories) and detected with diaminobenzidine (DAB) (Vector Laboratories). For the detection of Htt^exp ^protein aggregation, sections were stained with anti-Htt monoclonal antibody (1:200 dilution: Millipore), amplified and detected as before, and counter-stained with cresyl violet. The stained sections were mounted with Dako Glycergel Mounting Medium (Dako) onto the slides. All quantitative stereological analyses were performed blindly with respect to the nature of slices (genotype and drug feeding) using the Stereo Investigator setup and software (MicroBrightField Inc., Williston, VT). All quantitative stereological analyses were performed blindly with respect to the nature of slices (genotype and drug feeding) using Stereo Investigator. The grid size was set to 450 × 450 μm, and the counting frame was set to 50 × 50 μm. The average slice thickness after histological processing was determined to be 22 μm.

### Pathological analysis

Before perfusion, a hind-leg with fresh skeletal muscle was taken from each terminally anesthetized mouse. After perfusion and removal of the brain, the mouse carcasses were fixed in 10% buffered formalin until dissected. Representative sections of the heart, liver, lung and skeletal muscle samples from half of the animals in each group were obtained. Tissue was embedded in paraffin blocks, sectioned at 4 μm thickness, stained with hematoxylin and eosin, and coverslipped for light microscopic evaluation. The presence and degree of lymphocytic infiltrates in the four tissue types was semi-quantitatively graded using the following schema: 0, no inflammation; 1, rare, scattered, small collections of lymphocytes; 2, occasional scattered collections; and 3, frequent large collections.

### Statistical data analysis

For comparison between two or more groups, ANOVA with post hoc testing was used to statistically analyze data.

## Competing interests

The authors declare that they have no competing interests.

## Authors' contributions

XC carried out the feeding trial, motor skills assessments, *in vivo *assessment of cell death and drafted the manuscript. JW carried out the *in vitro *cell death assays. SL conducted the Ca^2+ ^imaging experiments. EH carried out the pathological analysis. CS participated in the drafting of the manuscript and data analysis. XC, JW, and SL performed the statistical analysis. IB conceived of the study, and participated in its design and coordination and helped to draft the manuscript. All authors read and approved the final manuscript.
